# Differential Nutrient Contents and Free Amino Acid Levels in Asymptomatic and Symptomatic Leaves of Huanglongbing-Affected Grapefruit Trees

**DOI:** 10.3390/plants14172756

**Published:** 2025-09-03

**Authors:** Aditi Satpute, Catherine Simpson, Mamoudou Sétamou

**Affiliations:** 1 Citrus Center, Texas A&M University-Kingsville, 312 N International Blvd., Weslaco, TX 78599, USA; catherine.simpson@ttu.edu (C.S.); mamoudou.setamou@tamuk.edu (M.S.); 2 Department of Plant and Soil Sciences, Texas Tech University, Bayer Plant Sciences Room 117A, 2911 15th Street, MS 42122, Lubbock, TX 79409, USA

**Keywords:** HLB progression, nutrient content, amino acids, sodium toxicity, symptoms, citrus diseases

## Abstract

Grapefruit (*Citrus* × *paradisi* Macfad.) is susceptible to Huanglongbing (HLB) disease, which prominently affects tree health and leads to a substantial loss of productivity. HLB-affected trees exhibit a nutritional imbalance expressed in either deficiencies or toxicities of the essential minerals required for plant growth, as well as changes in the production of plant metabolites. Hence, understanding foliar nutritional and metabolite fluctuations as HLB-elicited symptoms progress can assist growers in improving tree health management strategies. This study evaluated changes in foliar nutrient and phloem sap amino acid concentrations of HLB-affected grapefruit trees showing a mixed canopy of HLB-induced blotchy mottle and asymptomatic mature leaves. The trees used in our experiment were fruit-bearing seven-year-old grapefruit trees (cv ‘Rio Red’ on sour orange rootstock) grown in South Texas. Two types of foliage from HLB-affected trees were studied, (a) HLB-symptomatic and confirmed *Candidatus* Liberibacter asiaticus (CLas)-positive (IS) and (b) CLas-negative and HLB-asymptomatic (IA) mature leaves, which were compared to asymptomatic and CLas-free mature foliage from healthy trees (HY) in terms of their leaf nutrient and phloem sap amino acid contents. Hierarchical clustering based on leaf nutrient contents showed that 70% of IA samples clustered with HY samples, thus indicating that the levels of some nutrients were statistically similar in these two types of samples. The concentrations of the macronutrients N, Ca, Mg, and S and the micronutrients Mn and B were significantly reduced in HLB-symptomatic (IS) leaves, as compared to their IA and HY counterparts, which did not show statistically significant differences. Conversely, leaf Na concentration was approximately two-fold higher in leaves from HLB-affected trees (IA and IS) independent of symptom expression as compared to leaves from healthy trees. Significantly higher concentrations of glutamine and the S-containing amino acids taurine and cystathionine were observed in the IS leaves relative to the phloem sap of IA leaves from HLB-affected trees. In contrast, the phloem sap of IA (14%) and IS (41%) leaves from HLB-affected trees exhibited lower levels of γ-amino butyric acid (GABA) as compared to HY leaves. The results of this study highlight the changes in leaf nutrient and phloem sap amino acid profiles following CLas infection and HLB symptom development in grapefruit, and we discuss these results considering the strategies that growers can implement to correct the nutritional deficiencies and/or toxicities induced by this disease.

## 1. Introduction

Grapefruit, *Citrus* × *paradisi* Macfad., is an economically important crop in all the major citrus-producing states of the U.S. Commercial citrus production in Texas is concentrated in the southern-most region of the state, which is also known as the Lower Rio Grande Valley (LRGV), in which approximately 70% of total citrus acreage consists of grapefruit cv. ‘Rio Red’. Since its first detection in 2005, Huanglongbing (HLB), or citrus greening disease, has caused a substantial decline in citrus production in Florida, and the disease is threatening the sustainability of other major citrus industries of California and Texas [1]. Putatively caused by the obligate phytopathogen and phloem-inhabiting alpha-proteobacteria *Candidatus* Liberibacter spp., HLB is currently an incurable disease [2]. In the U.S., HLB is caused by *Candidatus* Liberibacter asiaticus (CLas) transmitted and spread by the Asian citrus psyllid, *Diaphorina citri* Kuwayama (Hemiptera: Psyllidae) [3]. Extensive screening efforts have identified genetic diversity in HLB response among citrus and related genera. Most of the commercially important citrus species are widely reported to be permissive for CLas multiplication and susceptible to HLB disease, including grapefruit, sweet orange (*Citrus sinensis* (L.) Osbeck), lemon (*Citrus* × *limon* (L.) Burm. f var. *limon*), lime (*Citrus* × *aurantiifolia* (Christm.) Swingle), and mandarin (*Citrus reticulata* Blanco.) [4,5].

In permissive hosts, CLas multiplication and HLB symptom development lead to major metabolic changes that weaken the tree [6]. HLB-affected trees exhibit a host of symptoms, including the loss of fibrous roots [7], blotchy mottled and chlorotic leaves with corky veins [8], small-sized and lopsided fruit [9], excessive fruit drop and off-flavor juice [10], and a steady decline in tree health and productivity. The underlying mechanisms of HLB symptom development in permissive hosts have been elucidated using advanced molecular biology techniques. At the nucleic acid level, HLB-elicited symptomatic plant organs show a differential reprogramming of gene transcription [11,12] and changes in protein translation [13] and metabolite content [14]. Metabolic disorders of various sugars and carbohydrates were observed in HLB-affected symptomatic leaves. Specifically, the increased activity of starch anabolism and the accumulation of starch granules were found to be associated with chlorophyll disruption and the creation of typical asymmetric discoloration in HLB-affected foliage, which is also known as blotchy mottle [8,9]. CLas infection leads to the overproduction of callose polymers that cause phloem plugging and affect the vascular anatomy [15], obstructing nutrient flow from source to sink organs in affected plants [16]. It is commonly reported that CLas infection and HLB symptom development reduce the concentrations of many microelements in citrus leaves. Similarly, the levels of key macronutrients, including nitrogen (N), calcium (Ca), and magnesium (Mg), significantly decrease with CLas infection. Altogether, imbalances in the plant ionic pool lead to nutritional deficiencies or toxicities due to either a reduction in uptake or the accumulation of certain nutrients in HLB-affected trees [17,18,19].

Some wild relatives in the subfamily Aurantioideae of *Rutaceae* exhibit natural resistance or strong tolerance to CLas multiplication, thus offering valuable breeding resources. Conventional breeding, somatic hybridization, and genetic engineering can be key strategies for developing HLB-resistant citrus. Australian finger lime genetics, for example, have been leveraged to produce novel hybrid rootstocks and scions with increased HLB tolerance [20,21,22]. Recent work incorporates genes from resistant wild relatives or uses genome editing (e.g., CRISPR) to introduce disease resistance traits [23]. Transcriptome analyses reveal that resistant or tolerant accessions activate stronger basal immune responses and stress-related pathways compared to susceptible varieties. Non-permissive species either are resistant to CLas multiplication or only transiently support bacterium replication, and they mostly include non-citrus genera such as *Clausena*, *Eremocitrus*, *Glycomis*, and *Murraya* [4,24]. Despite advances in breeding, no commercially viable, universally HLB-resistant citrus cultivar exists. The complexity of citrus genetics and the lack of robust field screening systems for HLB resistance present ongoing challenges.

As a cure for HLB is still elusive, disease mitigation strategies via the use of enhanced nutritional programs to correct deficiencies and prevent toxicities have been investigated to help growers to prolong the productive life of HLB-affected citrus trees [25,26]. Such an approach requires an in-depth understanding of the nutritional and metabolite changes induced by HLB. To date, there is no known study reporting the changes induced by CLas infection and HLB symptom development in grapefruit in Texas. To fill this knowledge gap and develop enhanced nutritional management strategies for HLB-affected trees in Texas, where the disease is currently endemic [3,27], this study was conducted to evaluate leaf nutritional and amino acid profiles following CLas infection and disease symptom development.

## 2. Materials and Methods

### 2.1. Plant Material and Treatment Categorization

This study was conducted in a seven-year-old grapefruit (cv. ‘Rio Red’ grafted onto sour orange rootstock) grove located in LRGV, TX. In this experiment, HLB-positive and healthy ‘Rio Red’ trees were selected for analyses of leaf nutrients and amino acids in phloem sap. The trees in the respective HLB categories were monitored for CLas presence using quantitative polymerase chain reaction (qPCR) diagnosis and foliar symptoms for more than 3 years prior to being sampled for this study. The HLB-positive trees had been confirmed as CLas-positive for at least two years, while ‘HLB-negative source trees’ were non-symptomatic and CLas-free. HLB-positive source trees showed a mixed canopy of typical HLB symptoms, including asymmetrical blotchy mottling on leaves and corky and yellow veins [8]. To test the differential effects of the presence or absence of HLB symptoms on leaf nutrient contents and the levels of amino acids in phloem sap, HLB-positive source trees were used to collect both HLB-symptomatic (IS) and -asymptomatic (IA) mature leaves (Table 1). Asymptomatic mature leaves from HLB-positive source trees were CLas-negative, while symptomatic samples were confirmed to be CLas-positive. Additionally, HLB-asymptomatic mature leaves collected from healthy trees confirmed as CLas-negative were used as the healthy control (HY) (Table 1). All samples were collected from five to six trees as biological replicates.

All trees in this study were managed following standard commercial practices, including flood irrigation, fertilization, and pest and disease management, as needed. The yearly fertilization program was similar and included two equal split applications of nitrogen as urea in February and April at a total rate of 168 kg N/ha, as well as three foliar applications (February, April, and July) of a blend of macro- and micronutrients. This water-soluble blend (Microplex, Miller Chemical & Fertilizer, LLC, Hannover, PA, USA) contained 5.43% magnesium, 0.5% zinc, 0.05% cobalt, 1.5% copper, 4% iron, 4% manganese, 0.1% molybdenum, and 1.5% zinc.

### 2.2. Foliage Sampling for CLas Diagnosis, Phloem Extraction, and Nutrient Analysis

Visual surveys of the experimental grove were conducted to identify HLB-affected trees twice a year (November and April) over a three-year period from 2013 to 2015. The canopy of each individual tree in the approximately 2-hectare grapefruit block (N 26°08′12.12″, W 97°56′46.97″, 23.5 m altitude) was thoroughly examined by two surveyors, with one located on each side of the tree canopy. Any HLB-symptomatic mature leaf was collected for a PCR confirmatory test in the laboratory for CLas presence. Six HLB-affected trees included in the present study were identified and monitored for at least two years prior to sample collection after fruit set in May–June 2015. HLB-symptomatic and -asymptomatic non-fruit-bearing twigs (each containing 10–12 matured leaves) were identified and labeled from each of the six selected HLB-positive source trees. Similarly, twigs with at least 10 mature leaves were randomly selected and flagged from healthy trees.

Two leaves were randomly collected per marked twig and pooled for a real-time PCR quantification cycle for CLas detection. The PCR test was conducted at the Diagnostic Lab at Texas A&M University-Kingsville, Citrus Center, Weslaco, TX, following a USDA protocol. Briefly, according to Li et al., primers [28,29] were used to amplify the conserved 16S rRNA region of CLas up to 40 cycles. Only upon qPCR confirmation was it that the individual twigs were categorized into the IA, IS, and HY treatments for downstream amino acid quantification and nutrient analysis.

### 2.3. Determination of Amino Acid Content and Foliar Nutrients

The twigs (ca. 10–15 cm in length) from each treatment group were excised and individually inserted into tubes containing ethylenediaminetetraacetic acid (EDTA) for phloem sap exudation, as described by King and Zeevaart [30] and Sétamou et al. [24]. Briefly, twigs with mature leaves were excised at the point of attachment to the branch using sterilized pruning shears and immediately immersed in 30 mL of 20 mM EDTA solution in plastic vials. The vials were then covered with moist paper towels and transported on dry ice to the laboratory to maintain sample integrity. Samples were agitated at 100 rpm on a table shaker (Innova 2300 Platform Shaker, New Brunswick Scientific, Enfield, CT, USA) in a dark, temperature (21 °C)-controlled room for 3 h. After phloem sap exudation and extraction, the twigs were removed, and the resulting EDTA–phloem sap extracts were transferred into sterile 50 mL centrifuge tubes and stored at −80 °C until further processing.

The EDTA–phloem exudate was freeze-dried (Millrock Bench-Top Freeze-Dryer BT48, Millrock Technology, Kingston, NY, USA), and the dried powder was analyzed for amino acid content at the University of Missouri-Columbia Experimental Station Chemical Laboratories [24]. The freeze-dried samples were re-suspended in 2 mL of 0.01 N HCl, and a 50 μL aliquot of each solution was analyzed for amino acids using a High-Speed Amino Acid Analyzer (Model: L-8900, Hitachi Ltd., Tokyo, Japan) and by following the methods previously described by Deyl [31], Le Boucher [32], and Fekkes [33].

For nutrient analysis, leaves were removed from the same experimental twigs, washed in 1% HCL solution, rinsed twice in distilled water, and air-dried in the laboratory. Leaves were grouped into two biological replicates of 5–6 leaves each per tree, for a total of 10 samples per treatment, and they were then sent to the Texas A&M University Soil, Water, and Forage testing lab, College Station, TX, for nutrient analysis.

### 2.4. Statistical Analysis

A one-way analysis of variance (ANOVA) was used to compare the leaf nutrient content and total amino acid concentrations between the three types of tissue. Whenever significant F-values were obtained, treatment means were separated using Tukey’s HSD test. An agglomerative hierarchical cluster (AHC) analysis conducted with the Euclidian distance as the proximity type and the Ward’s agglomeration method with automatic entropy truncation was used to classify the different tissue types into a limited number of relatively homogenous groups according to their nutrient and free amino acid (FAA) levels. Clustering is a data analysis technique that consists of grouping similar data points together into clusters, in which data points within a cluster are more similar to each other than in other clusters. To prevent any bias and ensure that all features contribute equally to the different clusters, data were scaled using the Z-score. To minimize the heterogeneity within clusters, entropy truncation was used to prevent the ambiguous assignment of features to clusters [34]. A high entropy value of a data point suggests that it belongs almost equally to different clusters, while low entropy denotes a strong association with a specific cluster. Groupings of FAAs or nutrients were similarly performed using AHC, and a heat map was used to graphically represent the relationship between either FAA or nutrient profiles and the tissue types of the clusters generated. All analyses were performed using JMP Pro 17 statistical software (SAS Institute Inc., Cary, NC, USA) [35].

## 3. Results

### 3.1. Leaf Mineral Content of Grapefruit

Significant differences between the three types of grapefruit leaves were observed for the concentrations of most of the leaf mineral nutrients, as shown by the significant F-values in the one-way ANOVA (Table 2). IS leaves had significantly lower levels of N, Ca, Mg, S, Mn, and B compared to IA and HY leaves, but the concentrations of these nutrients were comparable in these two latter treatments (Table 2). Effect size also indicated that the treatment effect explained a large proportion (*η*^2^ > 0.14) of the variation observed in many nutrients (N, Ca, Mg, S, Mn, and B), while a moderate effect (0.06 ≤ *η*^2^ < 0.14) was observed for P, K, and Na.

The concentration of Fe was significantly higher in IA than IS leaves. In contrast, the Na level was substantially higher in HLB-affected leaves (IS and IA) as compared to HY leaves. The levels of P, K, Cu, and Zn did not significantly vary among treatments.

**Figure 1:**
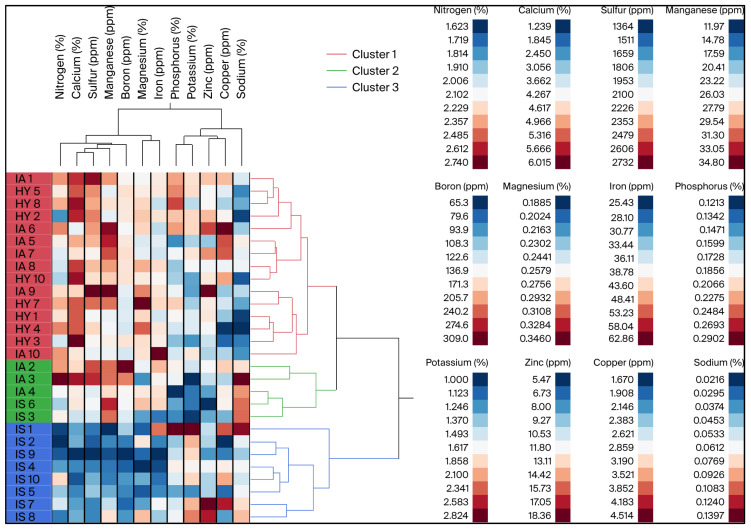
Two-way hierarchical cluster analysis (HCA). Heat map and dendrogram describing clustering of HY, IA, and IS based on leaf mineral nutrient concentration. HY: asymptomatic leaves from healthy trees; IA: asymptomatic leaves and CLas-negative leaves from HLB-affected trees; IS: infected symptomatic, i.e., symptomatic leaves and those confirmed as CLas-positive from HLB-affected trees. Three clusters are represented by red, green, and blue. Numbers following treatment ID represent biological replicates. Navy blue to dark red color scheme in legend box represents lowest to highest concentration gradient based on observed values for mineral nutrients collectively across treatments.

The agglomerative hierarchical clustering (AHC) analysis explained about 61.2% of the variation in leaf mineral nutrient contents and classified all samples in three clusters (Figure 1). The first cluster comprises all HY samples and 70% of IA samples, while the second cluster includes the three remaining IA samples and two IS samples. The third cluster consists of only IS samples, thus indicating that the ACH analysis separated the three types of samples well. The clustering of leaf nutrients resulted in the identification of three clades. Clade 1 contained the seven nutrients (N, Ca, S, Mg, Mn, Fe, and B) that were present in higher concentrations in HY leaves. Clade 2 contained the three nutrients that did not vary between treatments, while Clade 3 included Na and Cu, which tended to be present in higher concentrations in the leaves of trees positive for CLas (Supplement S1).

### 3.2. Phloem Sap Free Amino Acid Levels as Affected by HLB

The concentrations of amino acids in the phloem sap of the three types of tissue were quantified. A total of 22 proteogenic and non-proteogenic amino acids were detected from the phloem exudates of the twigs (Table 3). Five essential proteogenic amino acids, cysteine, isoleucine, leucine, methionine, and tryptophan, were below detectable levels in all treatments, whereas asparagine was only detected in the phloem sap of twigs from CLas-infected trees independent of symptom expression. The concentrations of taurine, cystathionine, and glutamine were significantly higher in the phloem sap of HLB-symptomatic twigs as compared to healthy twigs with a large effect size (*η*^2^ > 0.14), thus indicating a strong treatment effect (Table 3). In contrast, the level of GABA in the phloem of IS tissue was significantly lower than that of healthy tissue. No significant differences were recorded between the three types of tissue for the concentrations of most amino acids (Table 3). Similarly, the concentration of total amino acids measured in the extracted phloem did not vary with the type of tissue (F = 0.14; *p* = 0.87).

**Figure 2:**
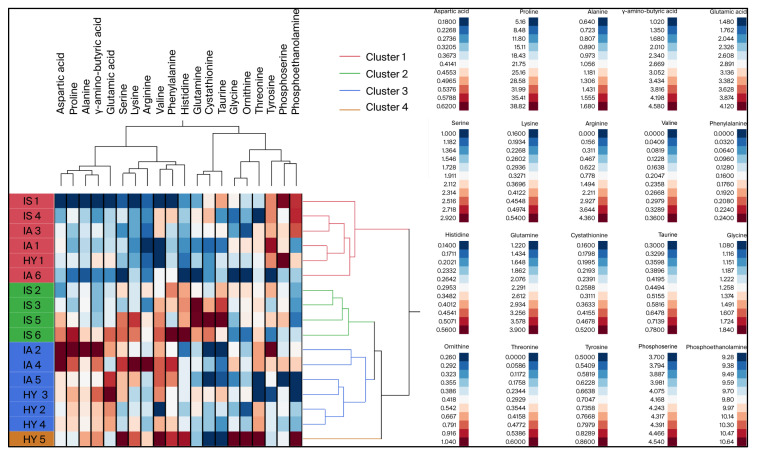
Two-way hierarchical cluster analysis (HCA). Heat map and dendrogram describing clustering of HY, IA, and IS samples based on phloem amino acid concentration (µg/g). HY: asymptomatic leaves from healthy trees; IA: asymptomatic leaves and CLas-negative leaves from HLB-affected trees; IS: infected symptomatic, i.e., symptomatic leaves and those confirmed as CLas-positive from HLB-affected trees. Numbers following treatment ID represent biological replicates. Four clusters are separated into red, green, blue, and orange. Navy blue to dark red color scheme in legend box represents lowest to highest concentration gradient based on observed values for amino acid concentration collectively across treatments.

Approximately 72% of the variation in phloem sap amino acid concentrations was explained by agglomerative clustering, which classified the twigs into three main clusters (Figure 2). While cluster 1 comprised twigs of the three types of HLB status, cluster 2 included only twigs of HLB-symptomatic leaves (IS), and cluster 3 contained 50% of both IA and HY twigs. The ACH analysis also grouped amino acids into four main clades (Figure 2). The first clades contained six amino acids that seem to be present in higher amounts in HY tissue, and their levels gradually declined with CLas infection and HLB symptom expression. The three amino acids (cystathionine, glutamine, and taurine) that were the most abundant in IS tissue were grouped in Clade 2. Clades 3 and 4 included, respectively, four and six amino acids, while Clade 4 included amino acids that were present in approximately equal amounts in the phloem sap of various tissues. The members of Clade 3 were the most abundant in HY compared to CLas-negative (IA and IS) tissues (Supplement S3).

## 4. Discussion

The leaf nutrient profiles and phloem sap amino acid concentrations of grapefruit exhibit significant alterations under CLas infection and Huanglongbing (HLB) symptom expression. These findings provide crucial insights into the physiological disruptions caused by *Candidatus* Liberibacter asiaticus (CLas), which is the bacterial pathogen responsible for HLB. A comparison between asymptomatic (HY and IA) and HLB-affected (IS) leaves revealed notable disparities in both macro- and micronutrient concentrations, as well as in free amino acid accumulation, highlighting the multifaceted impact of the disease on citrus metabolism. The HLB pathogen often induces varied nutrient disorders due to starch accumulation, physically plugging the transport tissues and impairing long-distance nutrient transport.

Specifically, the concentrations of key macronutrients, including N, Ca, Mg, and S, substantially decreased in HLB-symptomatic leaves, which is in line with previous observations in other citrus species [13,17,19,36,37]. Nitrogen is a critical component of chlorophyll and amino acids [38]; hence, the decrease in the N level in HLB-affected leaves to below adequate concentrations (Table 2) aligns with the observed blotchy mottle and chlorosis, which will reduce the photosynthetic activity of leaves from HLB-affected trees. The levels of calcium, which is vital for cell wall integrity and many physiological processes, were diminished by >50% in symptomatic leaves, potentially resulting from the restriction of nutrient uptake and movement [19]. This reduction in Ca concentration was reported as an important feature of HLB physiological disorders [39] and may contribute to the poor root development and reduced immunity typically observed in affected trees. Magnesium deficiency in plants could directly be associated with leaf discoloration as Mg is essential for photosynthesis and the activation of various enzymes, as well as being a major constituent of leaf chlorophyll [40,41]. Therefore, a decrease in Mg suggests compromised growth and reduced yields in HLB-affected trees. Sulfur is vital for amino acid, protein, and enzyme synthesis, and its level was reduced in IS leaves, thus possibly leading to the stunted growth and loss of yields, which are characteristics of HLB-affected trees.

The concentrations of some micronutrients such as boron, iron, and manganese were also reduced in HLB-symptomatic leaves. These elements play vital roles in enzymatic reactions, chlorophyll biosynthesis, and oxidative stress mitigation [42]. While boron deficiency leads to reduced and distorted fruits, iron and manganese deficiencies are known to cause leaf blotchiness and interveinal chlorosis, which are common visual symptoms of HLB [25]. In alkaline and calcareous soils, which prevail in South Texas, Fe and Mn tend to be deficient in leaves because the high pH reduces their solubility and uptake [43]. Hence, CLas infection will further exacerbate the symptoms caused by the deficiency of these nutrients.

In contrast to the reductions observed in the levels of N, S, Ca, Mg, Mn, Fe, and B, the CLas infection of grapefruit trees and HLB symptom development led to the accumulation of sodium in leaves. Both the IA and IS leaves collected from CLas-infected trees had at least 2-fold higher Na levels relative to asymptomatic leaf tissue in healthy trees. Sodium accumulation in citrus foliage may lead to toxicity in citrus trees which manifests as various symptoms, including premature leaf drop; the yellowing or browning of leaf margins and tips; stunted growth; and, in severe cases, bud, twig, and branch death, sometimes resulting in a witch’s broom appearance [44,45,46]. Sodium uptake by plants is selectively regulated by roots, and the accumulation of sodium is indicative of root stress [47]. One major impact of HLB is the loss of root hairs in affected trees [7]. In stressed plants, the leakage and accumulation of intra-cellular nutrients, specifically Na, are commonly observed, which also leads to Na’s competition with other cations such as K^+^ or Ca^2+^ through non-selective cation channels [48,49]. Therefore, nutrient stress in HLB-elicited chlorotic or blotchy mottled leaves could induce the differential regulation of Ca, Mg, K, and Na through cation transport competition. Hence, Na accumulation in CLas-infected and HLB-affected leaves may likely result from root damage which reduces the tree’s ability to exclude sodium. As Na competes with other essential cations (Ca, K, and Mg) for uptake by plant roots, its accumulation in HLB-symptomatic leaves of grapefruit may likely explain the concomitant reduction observed in the concentration of Ca and Mg (Table 2).

Apart from the Na level, the concentrations of all other nutrients were statistically comparable in IA and HY leaves, thus indicating that their reductions in IS leaves were solely due to HLB symptom development. Most IA leaves clustered with HY leaves, thus confirming the similarity of their nutrient profiles and pointing to the sectorial effects of HLB in citrus trees. These findings highlight the fact that an asymptomatic canopy of HLB-affected trees may still hold the optimal reserve of nutrients for normal physiological processes, thus opening avenues for the development of advanced canopy management tactics for HLB-affected groves. However, an increase in the Na level may not only be an indicator of CLas infection and HLB-related physiological disorders, but it can also significantly contribute to tree decline through toxicity. Hence, any enhanced nutritional program to keep HLB trees productive should also address the potential toxicity resulting from Na accumulation in leaves.

Despite the changes observed in multiple nutrients and specifically N and S, the shifts in the amino acid profile of HLB-affected twigs were limited to glutamine, taurine, γ-aminobutyric acid (GABA), and cystathionine. Glutamine, taurine, and cystathionine are frequently associated with plant stress responses and may accumulate as a result of disrupted protein synthesis, phloem plugging, or oxidative stress. Glutamine is a rich source of organic nitrogen that can be transported into the phloem [50,51]. Naturally, glutamine is found in higher quantities in CLas-permissive hosts such as ‘Rio Red’ grapefruit as compared to non-permissive citrus relatives [5], and this could explain the non-significant variation between HY phloem sap and HLB-positive (IA and IS) samples. However, the significant glutamine level variation between IA and IS makes it important to consider the possible utilization of glutamine as a source of N used by plants and pathogens as well in HLB-affected trees. The contrasting pattern of N and glutamine level changes in IS samples as compared to IA foliage in this study indicates that leaf N was remobilized, stored, or metabolized in the form of glutamine only at the symptomatic stage, which resulted in about 56% of its greater accumulation. This result is consistent with previous studies that reported increased levels of glutamine in the blotchy mottled leaf tissue of HLB-susceptible and -tolerant citruses grown in controlled conditions [52,53] or field-grown HLB-affected ‘Ruby Ray’ grafted onto sour orange [54]. In plant–pathogen interactions, glutamine accumulation is elicited as either a signaling strategy for plant defense or the remobilization of resources, whereas phytopathogens are found to be specialized in catabolizing N-rich amino acids to use them as an energy source, which leads to their increased levels in the vascular tissue [55]. However, no studies have elucidated the mechanism of glutamine accumulation in the presence of the CLas bacterium or blotchy mottled symptoms.

Taurine and cystathionine, which are both intermediate products of cysteine synthesis, are sulfur-containing non-proteogenic amino acids [56]. Their levels followed a similar trend as glutamine level changes in the IA and IS treatments in this study. Taurine and cystathionine phloem content was found to be significantly increased in the IS treatment as compared to the IA treatment. The reverse pattern of leaf S with respect to that of the levels of taurine and cystathionine in phloem sap in the IA and IS treatment indicated that foliar S was metabolized in taurine and cystathionine metabolite pathways. Taurine is abundant in mammalian cells and insects [57] and rarely studied in plants [58]. In previous research, taurine’s presence in CLas-permissive citruses was reported only in the young flush of non-infected citruses [5]. The current research highlights the detectable presence of taurine in mature HLB-negative and HLB-positive grapefruit leaves under field conditions. Its content in ‘Rio Red’ grapefruit, however, varied significantly only between IA and IS phloem sap, and symptomatic phloem sap was found to accumulate about 58% more taurine as compared to the phloem sap of asymptomatic leaves. The importance of taurine in citrus trees is rarely investigated, yet recent studies on the Mn toxicity of wheat seedlings [59] and salinity and Fe deficiency stress in *Pisum sativum* L. [60] highlighted the role of taurine in the detoxification of reactive oxygen species (ROS) through inducing endogenous antioxidant molecules such as hydrogen sulfide (H_2_S) and nitric oxide, suggesting that taurine could alleviate micronutrient toxicities or deficiencies. Moreover, S is also involved in stress tolerance [61,62] through tripeptides (glutathione), secondary metabolites (glucosinolates), and signaling molecules such as H_2_S, which is known to mitigate oxidative stress [63,64]. Altogether, the inverse patterns of S and taurine content in IA and IS ‘Rio Red’ grapefruit samples could indicate their relevance to the detoxification mechanism in response to HLB-induced oxidative stress. Indeed, the genes and proteins involved in antioxidant activity are widely reported to be upregulated in HLB-affected plant tissue [11,13]. Furthermore, the CLas genome does not appear to have genes for sulfur metabolism [65]. Therefore, in HLB-infected trees, taurine’s regulation in symptomatic leaves seems to be linked with micronutrient imbalances. In HLB-infected trees, deficiencies of Mn and Fe are often associated with loss of chlorophyll and yellowing of leaves [18], while research on taurine’s potential role in alleviating micronutrient-induced stress has been reported in wheat seedlings [59] and peas [60]. Altogether, these findings suggest that taurine regulation may have been influenced by differential micronutrient levels in CLas-infected leaves. More research is needed to understand taurine’s role in these processes.

The substantial reduction in GABA, which is a non-proteinogenic amino acid, suggests altered nitrogen metabolism in the phloem of HLB-affected twigs. Phloem GABA concentration gradually declined with CLas infection and HLB symptom development, and this observation is consistent with previous reports by Zhang et al. [66]. However, in Florida, GABA accumulation was observed in HLB-symptomatic sweet orange ‘Valencia’ leaves [6,67].

Based on the findings of this study, we discussed the possibility of using nutritional management strategies to correct deficiencies and mitigate any toxicity observed to maintain the productivity of HLB-affected grapefruit trees. By addressing key nutrient imbalances and mitigating stress, we believe that targeted nutritional management can help to support tree health and maintain better physiological function, even in the presence of CLas infection. While these strategies may not cure the trees or completely eliminate the negative impacts of the disease, they can contribute to maintaining tree productivity and fruit quality. Although there is no agreement among scientists and the commercial citrus industry about the usefulness of nutrition to mitigate HLB, in recent years, there has been a growing body of literature pointing to the benefits of enhanced nutrition in improving the health and maintaining the productivity of HLB-affected trees. Increased rates of Mn and Fe application have been reported to significantly enhance tree biomass and trunk diameter in HLB-affected greenhouse trees [68,69]. However, Morgan et al. [70] described a more complex interaction between enhanced nutrient application and yield response in HLB-affected trees. For instance, a specific rate of Mn supplementation substantially increased fruit yield, additional applications of Zn, B, and Mg did not affect the yield of HLB-affected sweet orange trees.

This study shows that the accumulation of specific stress-related amino acids, particularly glutamine and taurine, is a key hallmark of CLas infection. The observed changes in amino acids, such as elevated glutamine and taurine levels, may have practical applications beyond simply serving as markers of stress for timely HLB management strategies. These metabolites can potentially be evaluated as their exogenous application may hold promise for supporting plant stress responses and improving tolerance to HLB. This approach requires further validation.

Although CLas infection and HLB symptom development have been reported to reduce the proportion of essential amino acids in the leaves and phloem sap of citrus trees [66], there is limited research on the effects of the exogenous application of amino acids on mitigating HLB disease. Amino acids can enhance citrus health by acting as biostimulants that improve nutrient absorption, increase stress resistance, boost metabolism, and improve fruit quality. In lime, induced resistance to citrus canker was reported with the application of the amino acid methionine, thus leading to a decrease in necrotic lesions caused by the disease [71]. Furthermore, the foliar application of a blend of seaweed extract and amino acids was reported to improve the vegetative growth, yield, and fruit quality of mandarin and sweet orange [72]. However, to our knowledge, the effects of the exogenous application of amino acids remain to be explored.

A follow-up study addressing the effects of micronutrient and amino acid supplementation to correct the deficiencies observed in HLB-affected trees on fruit yield and quality is underway, and the preliminary results are promising.

Collectively, these findings underscore the profound metabolic reprogramming induced by HLB in citrus leaves and phloem sap. The disruption of nutrient homeostasis and the accumulation of stress-related amino acids reflect the systemic nature of CLas infection and its interference with both primary and secondary metabolism. From a management perspective, these insights highlight the importance of targeted nutrient supplementation and stress mitigation strategies to support tree health in HLB-endemic areas. Importantly, in addition to correcting the nutrient deficiencies of key elements such as N, Ca, Mg, and S, and some micronutrients (e.g., B, Fe, and Mn), alleviating Na toxicity symptoms should be an important component of these HLB mitigation strategies. Further studies are warranted to elucidate the causal mechanisms linking CLas infection to these biochemical changes and to explore the potential of an enhanced well-tailored citrus nutritional program as a component of integrated HLB management.

## 5. Conclusions

This study demonstrated that HLB leads to significant disruptions in nutrient homeostasis and amino acid metabolism in citrus trees, with clear differences observed between symptomatic and asymptomatic leaf tissues from CLas-infected trees. Our findings highlight that asymptomatic leaves of CLas-infected trees can maintain nutrient and amino acid profiles similar to that of healthy controls, thus indicating the importance of asymptomatic canopy patches in CLas-infected citrus trees to improve tree vigor through nutritional programs and possibly amino acid supplementation. Based on these results, future research should focus on refining nutritional management approaches as part of a broader integrated HLB control strategy. This includes optimizing nutrient supplementation tailored for different HLB severity levels, exploring the potential benefits of exogenous amino acid applications, and evaluating their interaction with other disease management practices. Integrating precise nutritional strategies with existing cultural, chemical, and biological controls may offer a more sustainable and effective approach to mitigating HLB’s impact on citrus health, fruit quality, and yield.

## Figures and Tables

**Table 1:** Sample identification.

Tree ID	Treatment	Description (Tree CLas Status–Foliar Status)	Source Tree HLB Status	Flush–Cq ^x^	Analyses Performed ^z^
IS 1	IS	Infected–symptomatic	Positive	24.2	AA, MN
IS 2	IS	Infected–symptomatic	Positive	26.3	AA, MN
IS 3	IS	Infected–symptomatic	Positive	23.4	AA, MN
IS 4	IS	Infected–symptomatic	Positive	23.9	AA, MN
IS 5	IS	Infected–symptomatic	Positive	28.4	AA, MN
IS 6	IS	Infected–symptomatic	Positive	29.5	AA
IA 1	IA	Infected–asymptomatic	Positive	ND ^y^	AA, MN
IA 2	IA	Infected–asymptomatic	Positive	ND	AA, MN
IA 3	IA	Infected–asymptomatic	Positive	ND	AA, MN
IA 4	IA	Infected–asymptomatic	Positive	ND	AA, MN
IA 5	IA	Infected–asymptomatic	Positive	ND	AA, MN
IA 6	IA	Infected–asymptomatic	Positive	ND	AA
HY 1	HY	Healthy	Negative	ND	AA, MN
HY 2	HY	Healthy	Negative	ND	AA, MN
HY 3	HY	Healthy	Negative	ND	AA, MN
HY 4	HY	Healthy	Negative	ND	AA, MN
HY 5	HY	Healthy	Negative	ND	AA, MN

IS: infected symptomatic, i.e., symptomatic leaves and those confirmed as CLas-positive from HLB-affected trees; IA: asymptomatic leaves and those confirmed as CLas-negative from HLB-affected trees; HY: asymptomatic leaves from healthy trees. Leaf ^x^ Cq values represent the qPCR cycle threshold for CLas detection in IS leaves. The Cq values of IS leaves are derived from a pooled sample of 2 leaves. ^y^ ND: not detected (Cq values were ≥37). ^z^ AA: amino acid; MN: leaf mineral nutrient content.

**Table 2:** Mean (±SE) nutrient content (% or ppm) of leaf tissue in three types of ‘Rio Red’ grapefruit leaf samples.

Nutrients	Code	HY ^1^	IA ^1^	IS ^1^	*F*-Value	*p*-Value	Eta-Squared ^4^ (*η*^2^)	Survey Range ^2^
Nitrogen (%)	N	2.15 ± 0.06 a	2.28 ± 0.07 a	1.88 ± 0.06 b	9.85	0.0006	0.4217	2.0–2.6
Phosphorus (%)	P	0.20 ± 0.01	0.18 ± 0.01	0.18 ± 0.01	0.98	0.3896	0.0674	0.13–0.5
Potassium (%)	K	1.58 ± 0.09	1.52 ± 0.09	1.79 ± 0.17	1.28	0.2955	0.0864	0.8–2.2
Calcium (%)	Ca	5.19± 0.27 a	4.84 ± 0.17 a	2.64 ± 0.28 b	30.20	<0.0001	0.6911	1.5–5.5
Magnesium (%)	Mg	0.28 ± 0.01 a	0.26 ± 0.01 a	0.23 ± 0.01 b	7.49	0.0022	0.3647	0.3
Sulfur (ppm)	S	2228.37 ± 69.6 a	2365.87 ± 80.3 a	1687.53 ± 69.7 b	24.28	<0.0001	0.4248	1500–5000
Sodium (%)	Na	0.03 ± 0.002 b	0.07 ± 0.01 a	0.08 ± 0.01 a	9.97	0.0006	0.0522	NA ^3^
Copper (ppm)	Cu	2.53 ± 0.18	3.21 ± 0.23	2.77 ± 0.28	2.25	0.1248	0.2173	5.0–20
Manganese (ppm)	Mn	26.25 ± 0.66 a	30.78 ± 0.92 a	20.73 ± 2.32 b	11.38	0.0003	0.1428	25–200
Zinc (ppm)	Zn	12.16 ± 0.31	12.44 ± 1.03	10.90 ± 1.25	0.72	0.4983	0.4574	25–150
Iron (ppm)	Fe	38.35 ± 1.43 ab	42.65 ± 2.5 a	33.85 ± 2.69 b	3.75	0.0366	0.6513	60–200
Boron (ppm)	B	142.68 ± 5.89 a	168.84 ± 18 a	96.61 ± 6.99 b	9.86	0.0006	0.4220	30–100

^1^ HY: asymptomatic leaves from healthy trees; IA: asymptomatic leaves and CLas-negative leaves from HLB-affected trees; IS: infected symptomatic, i.e., symptomatic leaves and those confirmed as CLas-positive from HLB-affected trees. Means (*n* = 10) followed by different letter within row are significantly different (*p* < 0.05, Tukey’s HSD test). ^2^ Survey range of grapefruit leaf nutrient content in Texas is provided by Texas A&M AgriLife Extension Soil, Water, and Forage Testing Laboratory and is specific to Texas’s grapefruit production conditions. ^3^ NA: not applicable. ^4^ Effect size was calculated as Eta-squared (*η*^2^) by dividing sum of square of effect by total sum of square of model, and it measures total variance in nutrient explained by treatments.

**Table 3:** Mean amino acid concentration (µg/g) of healthy and Huanglongbing (HLB)-affected asymptomatic and symptomatic leaves analyzed via HPLC.

Amino Acids	Healthy (HY ^1^)	Infected–Asymptomatic (IA ^1^)	Infected–Symptomatic (IS ^1^)	F Ratio	*p* Value	Eta-Squared ^2^ (*η*^2^)
Serine	1.98 ± 0.26	1.84 ± 0.25	1.92 ± 0.22	0.0914	0.9132	0.013
Glycine	1.34 ± 0.12	1.23 ± 0.05	1.22 ± 0.03	0.8778	0.4374	0.111
Alanine	1.12 ± 0.06	1.08 ± 0.13	0.98 ± 0.08	0.581	0.5722	0.077
Cysteine	—	—	—	—	—	—
Valine	0.18 ± 0.08	0.22 ± 0.05	0.21 ± 0.04	0.1283	0.8806	0.018
Leucine	—	—	—	—	—	—
Aspartic Acid	0.41 ± 0.01	0.47 ± 0.05	0.36 ± 0.05	1.5633	0.2439	0.183
Asparagine	—	1.15 ± 0.60	0.47 ± 0.47	—	—	
Threonine	0.34 ± 0.10	0.29 ± 0.06	0.25 ± 0.05	0.3492	0.7112	0.048
Methionine	—	—	—	—	—	—
Isoleucine	—	—	—	—	—	—
Lysine	0.33 ± 0.04	0.33 ± 0.05	0.32 ± 0.05	0.0217	0.9785	0.003
Tyrosine	0.66 ± 0.04	0.73 ± 0.05	0.71 ± 0.04	0.5884	0.5684	0.078
Phenylalanine	0.17 ± 0.02	0.16 ± 0.02	0.15 ± 0.03	0.0799	0.9236	0.011
Tryptophan	—	—	—	—	—	—
Glutamic Acid	3.33 ± 0.25	2.80 ± 0.27	2.62 ± 0.28	1.7503	0.2097	0.200
Glutamine	2.14 ± 0.14 ab	1.83 ± 0.36 b	2.87 ± 0.36 a	4.7168	0.0272	0.403
Proline	23.84 ± 1.63	22.02 ± 4.75	19.74 ± 4.11	0.2634	0.7721	0.036
Arginine	0.66 ± 0.25	1.00 ± 0.68	0.65 ± 0.20	0.1910	0.8282	0.027
Ornithine	0.50 ± 0.08	0.36 ± 0.07	0.41 ± 0.07	0.8517	0.4477	0.108
Histidine	0.30 ± 0.06	0.22 ± 0.02	0.37 ± 0.05	2.9292	0.0866	0.295
Phosphoserine	4.20 ± 0.09	4.09 ± 0.08	4.22 ± 0.08	0.7753	0.4794	0.100
Phosphoethanolamine	9.82 ± 0.20	9.76 ± 0.19	9.83 ± 0.19	0.0419	0.9591	0.006
Taurine	0.41 ± 0.05 ab	0.36 ± 0.04 b	0.57 ± 0.04 a	6.5949	0.0096	0.485
Cystathionine	0.22 ± 0.03 b	0.20 ± 0.03 b	0.36 ± 0.03 a	9.9158	0.0021	0.586
γ-amino-butyric Acid	3.32 ± 0.35 a	2.86 ± 0.32 ab	1.93 ± 0.32 b	4.5925	0.0293	0.396
Urea	139.59 ± 24.66	126.50 ± 18.22	130.60 ± 82.32	0.0791	0.9243	0.011
Total Amino Acids (µg/g)	195 ± 23.37	179 ± 21.33	180 ± 21.33	0.1040	0.8699	0.020

^1^ HY: asymptomatic leaves from healthy trees; IA: asymptomatic leaves and CLas-negative leaves from HLB-affected trees; IS: infected symptomatic, i.e., symptomatic leaves and those confirmed as CLas-positive from HLB-affected trees. ^1^ Mean concentration is based on 6 biological replicates in each IS and IA treatment and 5 biological replicates in HY. Means followed by different letter within row are significantly different (*p* < 0.05, Tukey’s HSD test) “—”: undetected via HPLC. ^2^ Effect size was calculated as Eta-squared (*η*^2^) by dividing sum of square of effect by total sum of square of model, and it measures total variance in amino acid concentration explained by treatments.

## Data Availability

The datasets generated and/or analyzed during this study are available upon request.

## Supplementary Materials

The following supporting information can be downloaded at: https://www.mdpi.com/article/10.3390/plants14172756/s1, Supplement S1: Clustering of nutrients contributing to total explained variation in the three treatments; Supplement S2: Nutrient content at upper and lower confidence interval at 95% in three different treatments; Supplement S3: Clustering of amino acid contributing to total explained variation in the three treatments; Supplement S4: Amino Acid content at upper and lower confidence interval at 95% in three different treatments
