# Supplementary material for: Differential Nutrient Contents and Free Amino Acid Levels in Asymptomatic and Symptomatic Leaves of Huanglongbing-Affected Grapefruit Trees
Source: Plants (Basel). 2025 Sep 3;14(17):2756. doi: 10.3390/plants14172756 (PMC12430224; doi:10.3390/plants14172756)
Supplement: Supplementary file 1 [file plants-14-02756-s001.zip › plants-3760486-supplementary.pdf]

Supplement S1. Clustering of nutrients contributing to total explained variation in the three treatments

| Cluster[1] | Members[1] | RSquare with Own Cluster[2] | RSquare with Next Closest[3] | 1-RSquare Ratio[4] |
|---|---|---|---|---|
| 1 | Ca | 0.8420 | 0.0350 | 0.1640 |
| 1 | S | 0.8300 | 0.0020 | 0.1710 |
| 1 | N | 0.7060 | 0.0060 | 0.2960 |
| 1 | Mn | 0.6540 | 0.0030 | 0.3470 |
| 1 | B | 0.5650 | 0.0270 | 0.4470 |
| 1 | Mg | 0.4260 | 0.1860 | 0.7040 |
| 1 | Fe | 0.2830 | 0.0300 | 0.7400 |
| 2 | P | 0.7790 | 0.0270 | 0.2270 |
| 2 | K | 0.7190 | 0.0280 | 0.2890 |
| 2 | Zn | 0.3320 | 0.0430 | 0.6980 |
| 3 | Na | 0.6020 | 0.0440 | 0.4160 |
| 3 | Cu | 0.6020 | 0.0790 | 0.4320 |

HCA analysis explained [1]grouping of macro- and micro- nutrients in three clusters, [2]relation of individual nutrient with other nutrients in its cluster and [3]with nutrients in the next most similar cluster, and [4] relative similarities between a nutrient's own cluster and the next closest cluster.

Supplement S2: Nutrient content at upper and lower confidence interval at 95% in three different treatments

| **Nutrients** | **Code** | **HY** | | **IA** | | **IS** | |
|---|---|---|---|---|---|---|---|
| | | **Lower CI** | **Upper CI** | **Lower CI** | **Upper CI** | **Lower CI** | **Upper CI** |
| Nitrogen (%) | N % | 2.01 | 2.28 | 2.12 | 2.44 | 1.74 | 2.03 |
| Phosphorus (%) | P (%) | 0.18 | 0.22 | 0.16 | 0.20 | 0.15 | 0.21 |
| Potassium (%) | K (%) | 1.36 | 1.80 | 1.31 | 1.73 | 1.40 | 2.18 |
| Calcium (%) | Ca (%) | 4.57 | 5.81 | 4.44 | 5.25 | 1.99 | 3.29 |
| Magnesium (%) | Mg (%) | 0.26 | 0.30 | 0.25 | 0.28 | 0.20 | 0.26 |
| Sulfur (ppm) | (S) ppm | 2067.84 | 2388.91 | 2184.16 | 2547.57 | 1529.80 | 1845.25 |
| Sodium (%) | Na (%) | 0.03 | 0.04 | 0.05 | 0.09 | 0.06 | 0.10 |
| Copper (ppm) | (Cu)ppm | 2.12 | 2.94 | 2.70 | 3.72 | 2.14 | 3.39 |
| Manganese (ppm) | (Mn)ppm | 24.75 | 27.75 | 28.70 | 32.87 | 15.48 | 25.99 |
| Zinc (ppm) | (Zn) ppm | 11.44 | 12.88 | 10.11 | 14.76 | 8.06 | 13.73 |
| Iron (ppm) | (Fe) ppm | 35.10 | 41.59 | 37.00 | 48.30 | 27.77 | 39.93 |
| Boron (ppm) | (B) ppm | 129.36 | 156.01 | 128.16 | 209.53 | 80.79 | 112.43 |

HY: asymptomatic leaves from healthy trees; IA: asymptomatic leaves and CLas negative from HLB affected trees; IS: Infected symptomatic, i.e. symptomatic leaves and confirmed CLas positive from HLB affected trees. Lower and upper CI values denotes mean of individual nutrient level in respective treatment with a 95% interval at lower and upper limit

Supplement S3. Clustering of amino acid contributing to total explained variation in the three treatments.

| Cluster[1] | Members[1] | RSquare with Own Cluster[2] | RSquare with Next Closest[3] | 1-RSquare Ratio[4] |
|---|---|---|---|---|
| 1 | Proline | 0.877 | 0.379 | 0.198 |
| 1 | γ-amino-butyric Acid | 0.84 | 0.234 | 0.208 |
| 1 | Alanine | 0.821 | 0.293 | 0.252 |
| 1 | Glutamic Acid | 0.672 | 0.133 | 0.378 |
| 1 | Aspartic Acid | 0.756 | 0.357 | 0.38 |
| 1 | Phosphoserine | 0.185 | 0.152 | 0.961 |
| 2 | Cystathionine | 0.897 | 0.07 | 0.11 |
| 2 | Glutamine | 0.864 | 0.126 | 0.156 |
| 2 | Taurine | 0.836 | 0.121 | 0.186 |
| 3 | Glycine | 0.927 | 0.204 | 0.092 |
| 3 | Ornithine | 0.786 | 0.19 | 0.265 |
| 3 | Threonine | 0.498 | 0.086 | 0.549 |
| 3 | Phosphoethanolamine | 0.337 | 0.1 | 0.737 |
| 4 | Lysine | 0.855 | 0.321 | 0.213 |
| 4 | Serine | 0.81 | 0.411 | 0.322 |
| 4 | Valine | 0.657 | 0.247 | 0.456 |
| 4 | Phenylalanine | 0.667 | 0.389 | 0.545 |
| 4 | Arginine | 0.518 | 0.15 | 0.567 |
| 4 | Histidine | 0.555 | 0.23 | 0.578 |
| 5 | Tyrosine | 1 | 0.07 | 0 |

HCA analysis explained [1]grouping of amino acids in four clusters, [2]relation of individual amino acid with other amino acids in its cluster and [3]with amino acids in the next most similar cluster, and [4] relative similarities between a amino acid's own cluster and the next closest cluster.

Supplement S4: Amino Acid content at upper and lower confidence interval at 95% in three different treatments

| **Amino Acids** | **HY** | | **IA** | | **IS** | |
|---|---|---|---|---|---|---|
| | **Lower CI** | **Upper CI** | **Lower CI** | **Upper CI** | **Lower CI** | **Upper CI** |
| Serine | 1.43 | 2.54 | 1.33 | 2.34 | 1.42 | 2.43 |
| Glycine | 1.18 | 1.51 | 1.07 | 1.38 | 1.06 | 1.37 |
| Alanine | 0.90 | 1.35 | 0.87 | 1.29 | 0.77 | 1.18 |
| Cysteine | — | — | — | — | — | — |
| Valine | 0.06 | 0.30 | 0.10 | 0.33 | 0.10 | 0.33 |
| Leucine | — | — | — | — | — | — |
| Aspartic Acid | 0.31 | 0.52 | 0.38 | 0.56 | 0.27 | 0.45 |
| Asparagine | — | — | — | — | — | — |
| Threonine | 0.18 | 0.50 | 0.14 | 0.44 | 0.10 | 0.40 |
| Methionine | — | — | — | — | — | — |
| Isoleucine | — | — | — | — | — | — |
| Lysine | 0.22 | 0.43 | 0.24 | 0.43 | 0.22 | 0.42 |
| Tyrosine | 0.56 | 0.77 | 0.64 | 0.83 | 0.62 | 0.80 |
| Phenylalanine | 0.11 | 0.23 | 0.11 | 0.21 | 0.10 | 0.21 |
| Tryptophan | — | — | — | — | — | — |
| Glutamic Acid | 2.71 | 3.95 | 2.24 | 3.36 | 2.05 | 3.18 |
| Glutamine | 1.56 | 2.72 | 1.31 | 2.36 | 2.35 | 3.40 |
| Proline | 14.83 | 32.85 | 13.79 | 30.25 | 11.51 | 27.97 |
| Arginine | -0.37 | 1.70 | 0.05 | 1.94 | -0.29 | 1.60 |
| Ornithine | 0.33 | 0.67 | 0.20 | 0.52 | 0.25 | 0.56 |
| Histidine | 0.19 | 0.41 | 0.12 | 0.31 | 0.27 | 0.47 |
| Phosphoserine | 4.01 | 4.38 | 3.92 | 4.26 | 4.05 | 4.39 |
| Phosphoethanolamine | 9.38 | 10.25 | 9.36 | 10.16 | 9.43 | 10.23 |
| Taurine | 0.31 | 0.51 | 0.27 | 0.45 | 0.48 | 0.67 |
| Cystathionine | 0.15 | 0.28 | 0.14 | 0.26 | 0.30 | 0.42 |
| γ-amino-butyric acid | 2.57 | 4.07 | 2.18 | 3.54 | 1.25 | 2.62 |
| Urea | 86.71 | 192.47 | 78.22 | 174.77 | 82.32 | 178.87 |
| Total Amino acid (μg/g) | 144.75 | 245.01 | 133.73 | 225.25 | 135.10 | 226.62 |

HY: asymptomatic leaves from healthy trees; IA: asymptomatic leaves and CLas negative from HLB affected trees; IS: Infected symptomatic, i.e. symptomatic leaves and confirmed CLas positive from HLB affected trees.

Lower and upper CI values denotes mean of individual amino acid concentration in respective treatment with a 95% interval at lower and upper limit.
